# Adaboost-SVM-based probability algorithm for the prediction of all mature miRNA sites based on structured-sequence features

**DOI:** 10.1038/s41598-018-38048-7

**Published:** 2019-02-06

**Authors:** Ying Wang, Jidong Ru, Yueqiu Jiang, Jian Zhang

**Affiliations:** 1 College of Equipment control, Shenyang Ligong University, No.6, nanping middle road, hunnan new district, Shenyang, Liaoning 110159 China; 2 College of Computer and Control Engineering, Qiqihar University, No.42, Wenhua Street, Qiqihar, Heilongjiang 161006 China; 3 College of Light Industry and Textile, Qiqihar University, No.42, Wenhua Street, Qiqihar, Heilongjiang 161006 China; 4 College of information science and engineering, Shenyang Ligong University, No.6, nanping middle road, hunnan new district, Shenyang, Liaoning 110159 China

## Abstract

The significant role of microRNAs (miRNAs) in various biological processes and diseases has been widely studied and reported in recent years. Several computational methods associated with mature miRNA identification suffer various limitations involving canonical biological features extraction, class imbalance, and classifier performance. The proposed classifier, miRFinder, is an accurate alternative for the identification of mature miRNAs. The structured-sequence features were proposed to precisely extract miRNA biological features, and three algorithms were selected to obtain the canonical features based on the classifier performance. Moreover, the center of mass near distance training based on K-means was provided to improve the class imbalance problem. In particular, the AdaBoost-SVM algorithm was used to construct the classifier. The classifier training process focuses on incorrectly classified samples, and the integrated results use the common decision strategies of the weak classifier with different weights. In addition, the all mature miRNA sites were predicted by different classifiers based on the features of different sites. Compared with other methods, the performance of the classifiers has a high degree of efficacy for the identification of mature miRNAs. MiRFinder is freely available at https://github.com/wangying0128/miRFinder.

## Introduction

MicroRNAs (miRNAs) are an ~22 nucleotide (nt) long, conserved class of noncoding RNAs that play key regulatory roles in diverse biological processes and diseases^1,2^, especially cancers, by modulating the gene expression^3^. Therefore, identification of miRNAs has important significance for mining the association of miRNAs and diseases. In mammals, mature miRNAs derive from the hairpin in primary transcripts by two cleavages process: primary miRNA (pri-miRNA) is processed and canonically cleaved to precursor miRNA (pre-miRNA) by Drosha^4^, then pre-miRNA is exported by Exportin 5^5^ and cytoplasmically processed into a miRNA:miRNA* duplex by Dicer^6,7^. Afterwards, one strand of the duplex product becomes a mature miRNA and the other degrades^8–10^. In individual cases, both strands are entirely functional.

Because pre-miRNAs are conservative and have the typical features involving sequence, structure and free energy, much software has been development to identify the mature miRNAs from their pre-miRNAs based on computational methods. Table 1 displays the main algorithms and software for mature miRNA identification.

**Table 1:** The main algorithms and software for mature miRNA identification.

Algorithm	Species	classifiers	Web server
MatureBayes	Human, Mouse	Naïve Bayes	http://mirna.imbb.forth.gr/MatureBayes.html
MaturePred	Plant	SVM	http://nclab.hit.edu.cn/maturepred/
MiRpara	Human, plant	SVM	http://www.whiov.ac.cn/bioinformatics/mirpara
miRdup	Multiple species	Random forest	http://www.cs.mcgill.ca/_blanchem/mirdup/
miRRim2	Human		http://mirrim2.ncrna.org
mirExplorer	Human	Adaboosting	biocenter.sysu.edu.cn/mir/
Microprocessor SVM	Human	SVM	https://demo1.interagon.com/miRNA/
miRmat	vertebrate	Random forest	http://mcube.nju.edu.cn/jwang/lab/soft/MiRmat/
matPred	Human	SVM	

MatureBayes^11^ identifies the starting sites of mature miRNAs for mice and humans based on Naïve Bayes. As a result, the method finds that 7, 8 and 9 nt from the starting position have the typical biological features to distinguish mature miRNAs. Microprocessor SVM^12^, MiRpara^13^, MaturePred^14^ and matPred^15^ are developed using the SVM algorithm. Microprocessor SVM is proposed based on 686 features that are associated with sequence and structure, the accuracy of this method is 50%, and 90% of its predictions are within a 2 nt deviation. Due to too many features, the results are not easy to use for biological analysis. In addition, the forecasting ability for the 3′ arm of miRNA is relatively weak. MiRpara is trained for animals and plants, and upon filtering these features based on the greedy algorithm and the SPSS method, its accuracy reached 80%. MaturePred uses 160 features to identify mature plant miRNAs, and it extracts features from the miRNA:miRNA* duplex and the flanking region, and then 86 features were selected using the gain information algorithm. Thus, the method achieves a higher prediction performance. MiRRim2^16^ is designed based on phastcons and phylop scores, which are extracted from position 20 downstream and upstream the Drosha processing sites. Twelve submodels were trained according to the scores and base pair to predict mature human miRNA in their pre-miRNA. As a result, its sensitivity and positive predictive value exceeded 0.4. MiRmat and miRdup were designed based on the random forest algorithm. MiRmat^17^ is based on the molecular interaction and miRNA biogenesis for vertebrates and consists of two parts: Drosha and Dicer sites prediction. Using the random forest algorithm, MiRmat reached 77.8% and 92.8% accuracy, respectively, for the Dosha and Dicer sites. MiRdup^18^ predicted the mature miRNAs based on 100 features from five lineages of cleavage sites on the miRNA:miRNA* duplexes using the random forest algorithm integrated with adaptive boost (Adaboost). MirExplorer^19^ was designed using transition probability matrices and miRNA biogenesis vectors using the Adaboost method for 16 species, whereas with earlier methods, it obtained a specificity of 95.03% and a sensitivity of 93.71% on human data. In particular, matPred was proposed in our previous work. MatPred is a highly effective method for identifying mature miRNAs within novel pre-miRNA transcripts based on the SVM for humans. It significantly outperformed three other widely used methods. Recently, many up-to-date methods have been designed for pre-miRNA identification, and they have a detailed research on feature selection and algorithm optimization, such as iMiRNA-PseDPC^20^, gapped kernels^21^, methods based on structure status^22^ and miRNA-dis^23^. In addition, powerful web servers have been specifically used for extracting the features from the RNA sequences, such as repRNA^24^ and Pse-in-One^25^. They solve problems of various types in pre-miRNA identification and improve the performance of classification.

Although many methods are available for mature miRNA location prediction, they suffer from various limitations. The canonical biological features extraction from the characteristic hairpins directly affects the accuracy of algorithms. In the second structure of pri-miRNA, the default information always corresponds to ‘bulge’, ‘interloop’ and ‘multibranch loop’, which are associated with the change in free energy, but this biological feature receives little attention. Otherwise, the computational methods of mature miRNA identification remain the primary focus on the start sites of mature miRNAs, but the end sites of mature miRNAs have important functions, especially for the heterogeneous miRNAs finding that always occurs in the end of mature miRNAs^26–28^. More importantly, because the true and pseudo-mature miRNAs have a very high sequence similarity, the accuracy of mature miRNAs identification methods remains low, no deviation identification accuracy is less than 50%, and the 5 nt deviation identification accuracy is less than 90%.

In this study, we introduce a computational method, matFinder, that uses an AdaBoost-SVM algorithm to predict all the process sites of the mature miRNA in a pre-miRNA transcript. The structured-sequence features that focus on the default information in the secondary structure of pre-miRNAs are presented. For all the processing sites, corresponding models are trained based on their biological characters. More importantly, we design a mature miRNAs identification method using the AdaBoost and SVM algorithms. Because the AdaBoost algorithm adjusts the data set of SVMs based on the incorrectly classified samples, the accuracy of our method is promoted instead of that of the method based on SVM. In addition, comparison with existing tools suggests that MatFinder achieves the highest prediction accuracy.

**Figure 1:**
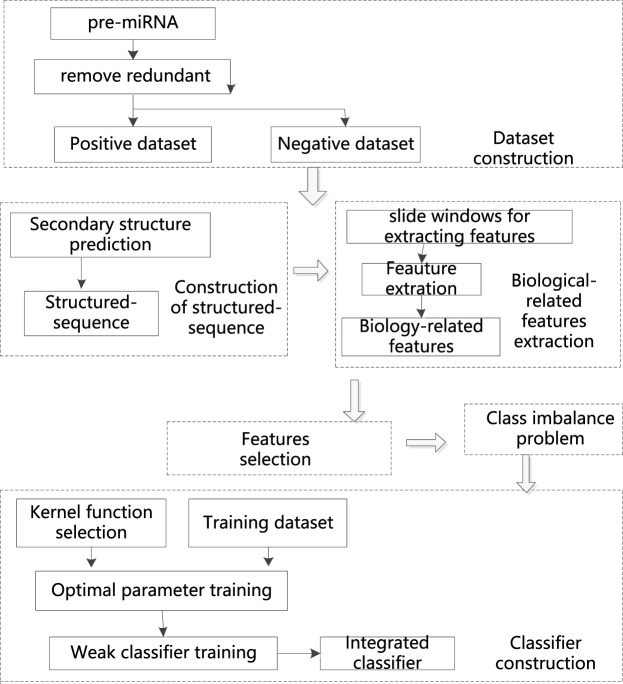
The schematic of the overall method. MiRFinder consists of five steps, namely, dataset construction, construction of structured-sequence, biological-related features extraction, feature selection, class imbalance problem and classifier construction. For features selection, three algorithms including information gain, chi-square and relief were investigated. For class imbalance problem, the AdaBoost algorithm was adopted. Moreover, for classifier construction, the AdaBoost and SVM were used. SVM is used as the weak classifier, and AdaBoost is used as the strong classifier.

The schematic of the overall method is illustrated in Fig. 1.

## Materials and Methods

### Data

**Figure 2:**
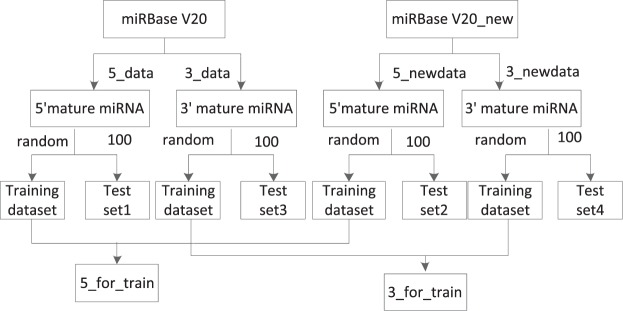
The process of dataset construction.

The training and test datasets were obtained from the miRBase V21^29^. The pre-miRNAs that have a secondary structure including multibranch loops were excluded. The process of dataset construction is shown in Fig. 2.

First, the human pre-miRNAs were downloaded from miRBase V20 and divided into 5_data and 3_data based on the location of the mature miRNAs. If the mature miRNA of pre-miRNA is located in the 5′ arm, then the pre-miRNA is divided into 5_data. Otherwise, if the mature miRNA of pre-miRNA is located in the 3′ arm, then the pre-miRNA is divided into 3_data. In a similar way, the pre-miRNAs that only belong to version 21, named miRBase V21_new, were downloaded from miRBase V21 and divided into 5_newdata and 3_newdata. Second, Test set1 and Test set2 for testing the 5′ arm mature miRNAs models were randomly selected from 5_data and 5_newdata. Between them, Test set2 was randomly selected from the newest dataset that only belongs to version 21. Moreover, Test set3 and Test set4 for testing the 3′ arm mature miRNAs models were randomly selected from 3_data and 3_newdata, and between them, Test set4 was randomly selected from the newest dataset that only belongs to version 21. Finally, except for the Test set, the other dataset was used to construct the training data. As a result, we constructed the 5_for_train and the 3_for_train datasets to train the models for identifying the 5′ arm and 3′ arm mature miRNAs, respectively. The 3_for_train and 5_for_train datasets consist of 1118 and 1071 pre-miRNAs sequences, respectively. Test set1, Test set2, Test set3 and Test set4 consist of 100 pre-miRNAs.

**Figure 3:**
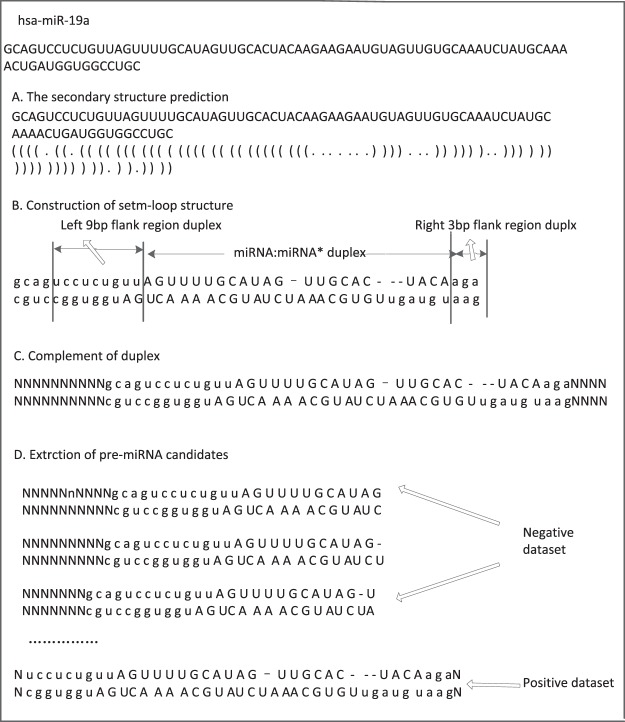
Structure-based dataset construction method.

The positive and negative datasets were constructed based on the above training and test datasets. Taking hsa-mir-19a as an example, the structure-based dataset construction method is shown in Fig. 3.

RNAfold^30^ was used to predict the secondary structure of each pre-miRNA, and then the stem-loop of each pre-miRNA was constructed based on the sequence and structure. Furthermore, the stem of the pre-miRNA was complemented by a 10 base pair (bp) double strand and a right base pair (bp). The double strand regions, which include 22 nt nucleotides in the 5′ arm, were extracted from the first to the last nucleotide using the sliding window method. Finally, the double strand, which was consistent with the starting position of the true mature miRNA, was selected as the positive example, whereas the other sequences were defined as the negative example.

### The extraction of feature set

**Figure 4:**
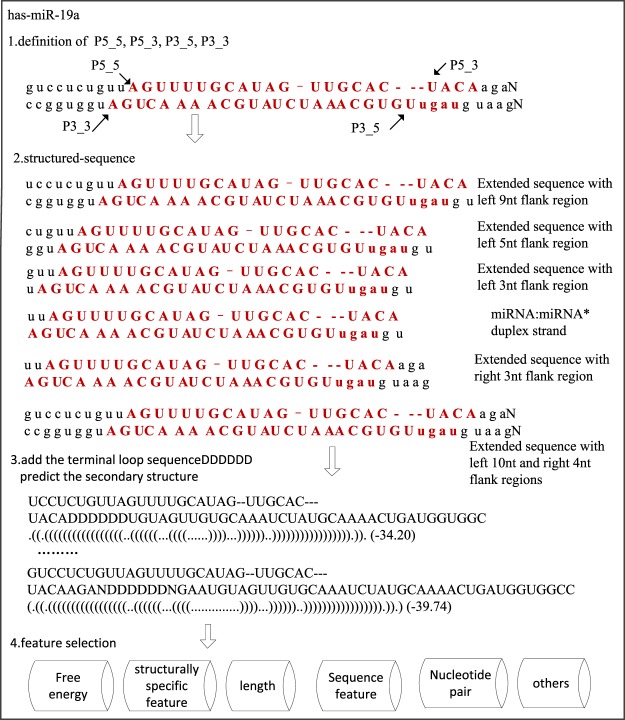
The process of the feature extraction.

Taking has-miR-19a as an example, the process of feature extraction is shown in Fig. 4. To extract the features of mature miRNAs, we predicted the secondary structure of each pre-miRNA using ViennaRNA software, and then constructed the structured-sequence based on the sequence, structure and the alignment information. Depending on the requirements of feature extraction, strand regions of different lengths were selected. Based on the sequence, structure, structured-sequence and these strand regions, the features were extracted.

The absence information, ‘−’, in the structured-sequence is important. It always means the change in free energy, sequence and structure associated with a ‘bulge’, ‘interloop’ and ‘multibranch loop’. To compare the performance of the structured-sequence with the absence information, we also extracted features without the absence information.

### Feature selection

The feature set of mature miRNA recognition includes hundreds of feature vectors, and such high-dimensional data can cause “dimension disaster”. The effective feature selection algorithm is an important part of data preprocessing. Feature selection can remove the uncorrelated redundant features, reduce the data set dimension, optimize and classify the high correlation characteristics, avoid dimension disaster in the machine learning method, and improve the generalization performance of the model. Importantly, by analyzing the characteristics of mature miRNAs, the typical characteristics of true and pseudo-mature miRNAs that differentiate mature miRNAs and other short nucleotide sequences are identified, which has important value and biological significance for miRNA recognition and its functional research.

In this paper, three feature selection algorithms are used to select the feature selection, namely, the information gain (IG), CHI (CHI) and relief algorithms. On the basis of a performance test, we selected the optimal feature selection algorithm.

1. The information gain (IG) selects characteristics by calculating the difference in information entropy. It defines class C_i_, and the information gain of characteristic t is defined as:
  1 $${\rm{IG}}({\rm{t}},{{\rm{C}}}_{{\rm{i}}})=-\,{\rm{P}}({{\rm{C}}}_{{\rm{i}}}){\rm{logP}}({{\rm{C}}}_{{\rm{i}}})+{\rm{P}}({\rm{t}}){\rm{P}}({{\rm{C}}}_{{\rm{i}}}|{\rm{t}}){\rm{logP}}({{\rm{C}}}_{{\rm{i}}}|{\rm{t}})+{\rm{P}}(\bar{{\rm{t}}}){\rm{P}}({{\rm{C}}}_{{\rm{i}}}|\bar{{\rm{t}}}){{\rm{logPC}}}_{{\rm{i}}}|\bar{{\rm{t}}})$$
  where t means t nonexistence. Then, the IG of t is defined as:
  2 $${\rm{IG}}({\rm{t}})={\sum }_{{\rm{i}}}{\rm{IG}}({\rm{t}},{{\rm{C}}}_{{\rm{i}}})$$
2. Chi-square statistic (CHI) selects features between the representation variables by calculating the correlation. The larger the statistical value of CHI is, the more important the feature is. For any type of C_i, the CHI value of characteristic t is:
  3 $${\rm{CHI}}({{\rm{C}}}_{{\rm{i}}},{\rm{t}})=\frac{{\rm{P}}({{\rm{C}}}_{{\rm{i}}},{\rm{t}}){\rm{P}}({\bar{{\rm{C}}}}_{{\rm{i}}},\bar{{\rm{t}}})-{\rm{P}}({\bar{{\rm{C}}}}_{{\rm{i}}},{\rm{t}}){\rm{P}}({{\rm{C}}}_{{\rm{i}}},\bar{{\rm{t}}})}{{\rm{P}}({{\rm{c}}}_{{\rm{i}}}){\rm{P}}({\rm{t}}){\rm{P}}({\bar{{\rm{C}}}}_{{\rm{i}}}){\rm{P}}(\bar{{\rm{t}}})}$$
3. Relief algorithm.

The relief algorithm selects the nearest neighbor according to the weight by calculating the distance between the samples. Set ${\rm{X}}=\{{{\rm{X}}}_{1},{{\rm{X}}}_{2},\ldots ,{{\rm{X}}}_{{\rm{n}}}\}$ as the sample dataset, and X_i_ = [X_i1_, X_i2_, …X_iN_]^T^ is the Nth character of the ith sample. The weight of the sample on each characteristic is defined as:

4 $${{\rm{W}}}_{{\rm{j}}}^{{\rm{i}}+1}={{\rm{W}}}_{{\rm{j}}}^{{\rm{i}}}-{\rm{diff}}\,({\rm{j}},{\rm{x}},{\rm{H}}\,({\rm{x}}))/m+{\rm{diff}}\,({\rm{j}},{\rm{x}},{\rm{M}}\,({\rm{x}}))/m$$

where H(x) and M(x) are the nearest neighbors of the same and different class of X, respectively, and diff is defined as:

5 $${\rm{diff}}\,({\rm{j}},{\rm{x}},{\rm{H}}\,({\rm{x}}))=\frac{{\rm{value}}\,({\rm{x}},{\rm{j}})-{\rm{value}}\,({\rm{H}}({\rm{x}}),{\rm{j}})}{{\rm{\max }}\,({\rm{j}})-\,{\rm{\min }}\,({\rm{j}})}$$

### Weak classifier training based on SVM with probability

Mature miRNAs identification identifies the true mature miRNAs from many short sequences that are constructed from one pre-miRNAs. Therefore, for each pre-miRNA, we cannot identify whether a short sequence is a mature miRNA, but the possibility exists that it is a mature miRNA, so here we introduced the SVM method based on probability to train a mature miRNA classifier. The SVM algorithm based on a probabilistic model is as follows:

Define mature miRNA training samples ${\rm{T}}=\{({{\rm{x}}}_{1},{{\rm{y}}}_{1}),({{\rm{x}}}_{2},{{\rm{y}}}_{2}),\ldots \ldots .,({{\rm{x}}}_{{\rm{N}}},{{\rm{y}}}_{{\rm{s}}})\}$, where x_i_ is the character value of the sample, y_s_ ∈ {1, −1}, and the number of samples is N, which includes N_a_ positive samples and N_b_ negative samples. Set each sample to having M characters, ${{\rm{x}}}_{{\rm{i}}}=\{{{\rm{x}}}_{{\rm{i}}}^{1},{{\rm{x}}}_{{\rm{i}}}^{2},\ldots \ldots ,{{\rm{x}}}_{{\rm{i}}}^{{\rm{M}}}\}$, and the class functions are defined as follows:

6 $${\rm{f}}({\rm{x}})=\sum _{{\rm{i}}=1}^{{\rm{n}}}\,{\propto }_{{\rm{i}}}\,{{\rm{y}}}_{{\rm{i}}}\, < \,{\rm{x}},\,{{\rm{x}}}_{{\rm{i}}}\, > \,+\,{\rm{b}}$$

where x_i_ is a character vector of some sample, x is the prediction sample, ∝_i_(0 ≤ ∝_i_ ≤ C) is a trainable coefficient, C is a penalty parameter, and <x, x_i_> is the inner product of x and x_i_. The kernel function is used to calculate the inner product, and it solves the problem of the data mapping from the original space to the high dimensional linear nonseparable problem. In particular, the radial basis function (RBF) is defined as follows:

7 $${\rm{K}}({{\rm{x}}}_{{\rm{i}}},{\rm{x}})\,= < \,{\rm{x}},\,{{\rm{x}}}_{{\rm{i}}}\, > =\,\exp \,(-\frac{{||{{\rm{x}}}_{{\rm{i}}}-{\rm{x}}||}^{2}}{2{{\rm{\delta }}}^{2}})$$

where δ is the conventional control parameter, which determines the weight of the feature.

The output of traditional SVM is binary. This means that for each point, it either belongs to a positive or negative class. In this way, for a pre-miRNA, all the candidate mature miRNA sequences on one arm are predicted to be pseudo-sequences, or the predicted mature miRNAs are greater than or equal to two sequences. Obviously, this is not biological. The mature miRNAs prediction needs to find the only mature miRNA in one arm of a pre-miRNA, so the SVM based on probability output was used to solve this problem.

For the sample χ, the posttest probability is:

8 $${{\rm{P}}}_{{\rm{i}}}={\rm{P}}({\rm{y}}=i|{\rm{\chi }}),\,{\rm{i}}=1,-\,1.$$

The sum of the probability of the sample belonging to two classes is 1. Therefore, the constraint conditions of equation (8) are:

9 $$\sum _{{\rm{i}}=1}^{{\rm{i}}=-1}\,{{\rm{P}}}_{{\rm{i}}}=1$$

10

Set r_ij_ to the probability estimate of two types of problems. According to (9), (10) proposes the following solution:

11 $${\rm{\min }}\,\frac{1}{2}{\sum }_{{\rm{i}}=1}^{{\rm{k}}}\,{\sum }_{{\rm{j}}\ne {\rm{i}}}\,{({{\rm{r}}}_{{\rm{ji}}}{{\rm{P}}}_{{\rm{i}}}-{{\rm{r}}}_{{\rm{ij}}}{{\rm{P}}}_{{\rm{j}}})}^{2},\,{\rm{s}}.{\rm{t}}.\,{\sum }_{{\rm{i}}=1}^{{\rm{k}}}\,{{\rm{P}}}_{{\rm{i}}}=1,\,{\rm{k}}=1,-\,1$$

(11) is calculated as follows:

12 $${\rm{\min }}\,\frac{1}{2}{{\rm{P}}}^{{\rm{T}}}{\rm{QP}}$$

where

13 $${{\rm{Q}}}_{{\rm{ij}}}=\{\begin{array}{ll}{\sum }_{{\rm{s}}:{\rm{s}}\ne {\rm{i}}}{{\rm{r}}}_{{\rm{si}}}^{2} & \,{\rm{if}}\,{\rm{i}}={\rm{j}}\\ -\,{{\rm{r}}}_{{\rm{ji}}}{{\rm{r}}}_{{\rm{ij}}} & {\rm{if}}\,{\rm{i}}\ne {\rm{j}}\end{array}\}$$

The matrix Q is a semipositive definite matrix, so equation (11) is a convex quadratic programming problem with linear constraints. If P is the optimal solution to the quadratic programming problem, the following conditions are met:

14 $$[\begin{array}{c}{\rm{Q}}\,{\rm{e}}\\ {{\rm{e}}}^{{\rm{T}}0}\end{array}][\begin{array}{c}{\rm{P}}\\ {\rm{b}}\end{array}]=[\begin{array}{c}0\\ 1\end{array}]$$

The solution to equation (11) can be obtained by solving linear equations.

Using the above method, the probability of each short sequence of a pre-miRNA is estimated, and the probability of the true mature miRNA is given. In the process of model training, it is necessary to optimize the two parameters of the planning factor C and the Gaussian width g. For the planning factor, if C → ∞, it is shown that the classification rules for satisfying all the constraint conditions will reduce the generalization ability and improve training complexity, so the C range must be as wide as possible to satisfy the classifier generalization performance. For parameter g, the optimization algorithm is used for adjusting, and the software grid py is used for training.

### Strong classifier constructed based on Adaboost

When SVM is combined with AdaBoost, on the one hand, the SVM algorithm makes up for the error in AdaBoost in processing high-dimensional data. On the other hand, the SVM algorithm follows the structure risk minimization principle, and the parameter optimization of the RBF_SVM classifier can improve the classification performance of a weak classifier. By selecting appropriate parameters C and g, it can avoid overfitting. In addition, the AdaBoost algorithm is also a process of data transfer in the integration process, and the research on the solution of the class imbalance problem is a direction worth exploring.

For the selection of data sets, the AdaBoost algorithm implements the training subset sample selection according to the continuous adjustment of the sample parameters. The method first sets the initial weight value, then adjusts the sample weight through the sample error rate during each progressive training process and adjusts the weight of the weak classifier accordingly. In the entire process, the weight of the wrong subsample is divided into emphasis to improve the recognition rate. Our method is based on AdaBoost, and the weak classifier adopts the SVM algorithm based on the probabilistic mode, and in each round of weak classifier training, the parameter optimization is performed. The Adaboost-SVM algorithm is described as follows:

Set the training dataset S = {(x_i_, y_i_)|i = 1,2, … n}, where x_i_ ∈ X is the mature miRNA sample, and y_i_ ∈ Y = {+1, −1} is the class of the samples.

Set the weight of x_i_ in the training set, which in the sample of training dataset S is ${{\rm{D}}}_{{\rm{t}}}({\rm{i}})\,$in round t, and the first round of sample weight is initialized to:

15 $${{\rm{D}}}_{1}=({{\rm{P}}}_{11},{{\rm{P}}}_{12}\ldots {{\rm{P}}}_{1{\rm{i}}}\ldots ,{{\rm{P}}}_{1{\rm{N}}}),{{\rm{P}}}_{11}={{\rm{P}}}_{12}=\ldots ={{\rm{P}}}_{1{\rm{N}}}=1/{\rm{N}}$$

We use the adjustable parameters of the SVM based on probability as a weak classifier. In the process of training, through the parameter adjustment, the optimal classification plane is selected, and for each of the pre-miRNA, a given probability of each sample that it is the true mature miRNA is given. The class of the candidate that has the largest probability is set as +1, the class of other candidates are set as −1, and the classifier G_t_(X): X → {−1, +1}.

Set the training round T.

Define the weight distribution of training set S:

16 $${{\rm{D}}}_{{\rm{t}}}=\{{{\rm{P}}}_{{\rm{t}}1},{{\rm{P}}}_{{\rm{t}}2},\ldots ,{{\rm{P}}}_{{\rm{tN}}}\}$$

where D_t_ is the vector that is constructed by all samples. From the training set, train the subset S_t_ based on the sample weight.

The error rate of the training subset is calculated by setting G_t_(X):{X → Y}. The sample error rate can be described as:

17 $${{\rm{e}}}_{{\rm{t}}}={\rm{P}}({{\rm{G}}}_{{\rm{t}}}({{\rm{x}}}_{{\rm{i}}}\ne {{\rm{y}}}_{{\rm{i}}}))=\sum _{{\rm{i}}=1}^{{\rm{n}}}\,{{\rm{P}}}_{{\rm{ti}}}\,{\rm{I}}({{\rm{G}}}_{{\rm{t}}}({{\rm{x}}}_{{\rm{i}}})\ne {{\rm{y}}}_{{\rm{i}}}))$$

The classifier weight is:

18 $${\propto }_{{\rm{t}}}=\frac{1}{2}\,\mathrm{log}\,\frac{1-{{\rm{e}}}_{{\rm{t}}}}{{{\rm{e}}}_{{\rm{t}}}}$$

Then, the weight of the sample can be update as:

19 $${{\rm{D}}}_{{\rm{t}}+1}=({{\rm{P}}}_{{\rm{t}}+1,1},{{\rm{P}}}_{{\rm{t}}+1,2}\ldots {{\rm{P}}}_{{\rm{t}}+1,{\rm{i}}}\ldots ,{{\rm{P}}}_{{\rm{t}}+1,{\rm{N}}})$$

where formula (19)can be shown as,

20 $${{\rm{P}}}_{{\rm{t}}+1,{\rm{i}}}=\frac{{{\rm{P}}}_{{\rm{t}}}{\rm{i}}}{{{\rm{Z}}}_{{\rm{t}}}}\exp (\,-\,{\propto }_{{\rm{t}}}\,{{\rm{y}}}_{{\rm{i}}}{{\rm{G}}}_{{\rm{t}}}({{\rm{x}}}_{{\rm{i}}})),\,{\rm{i}}=1,2,\ldots ,{\rm{N}}$$

In formula (20), z_t_ is defined as the weight of the next round t of the training set, which is a generalized constant defined as:

21 $${{\rm{Z}}}_{{\rm{t}}}=\sum _{{\rm{t}}=1}^{{\rm{T}}}\,{{\rm{P}}}_{{\rm{ti}}}\exp (\,-\,{\propto }_{{\rm{t}}}\,{{\rm{G}}}_{{\rm{t}}}({\rm{x}}))$$

Finally, the integrated classifier is defined according to the weak classifier ${{\rm{G}}}_{{\rm{t}}}({\rm{X}})$ and its weight:

22 $${{\rm{G}}}_{{\rm{x}}}={\rm{sign}}({\rm{f}}({\rm{x}}))={\rm{sign}}(\sum _{{\rm{t}}=1}^{{\rm{T}}}\,{\propto }_{{\rm{t}}}\,{{\rm{G}}}_{{\rm{t}}}({\rm{x}}))$$

### Improving classification by solving the class imbalance problem

The ratio of the positive dataset and negative dataset of these approaches is usually larger than 1:10. To improve the classification performance with respect to the imbalanced dataset including the positive and negative dataset, we designed the algorithm to solve the class-imbalance problem of mature miRNAs prediction.

Set the training dataset S = {S_neg_, S_pos_}. The K-means algorithm was used to cluster the positive samples. Given the negative dataset including n samples, define a threshold that is the number of clusters. Because the ratio of the positive and the negative dataset is 1:10, the threshold was set as 10. This means that the negative samples were divided into 10 clusters, namely, K_1_, K_2_, ……, K_10_, where K_i_ ∈ D, K_j_ ∈ D, K_i_ ∩ K_j_ = ϕ.

For any cluster K_i_, define k_m_ as the center of mass, which is obtained by calculating the average value of the features, and the distance of any sample k_n_ and the center of mass is defined as dist(k_n_, k_m_). The mass E is defined as the sum of all squared sample features in the cluster and the center of mass, as follows:

23 $${\rm{E}}=\sum _{{\rm{i}}=1}^{10}\,\sum _{{{\rm{k}}}_{{\rm{n}}}\in {{\rm{k}}}_{10}}\,{\rm{dist}}{({{\rm{k}}}_{{\rm{n}}},{{\rm{k}}}_{{\rm{m}}})}^{2}$$

By calculating the optimal distance distribution, the distance between samples in the cluster is minimized, but the distance between samples and other clusters is maximized and reaches the maximum degree of mutually independence between clusters.

The pseudocode for constructing the sample balance method of the center of mass near distance training based on the K-means algorithm is described as:

Algorithm: The sample balance method of the center of mass near distance training based on K-means

Input: negative dataset, cluster threshold

Output: negative subset

The processing flow:

Input negative dataset;

The threshold of the cluster;

Ten cluster masses were selected in the negative dataset.

The distances between all samples and the center of mass in the negative dataset were calculated and divided into 10 clusters.

The mean of each cluster was recalculated and was taken as the new center of mass of the cluster.

If the centers of mass are the same, the next step is taken. Otherwise, the center of mass is calculated circularly until the centers of mass are the same.

After determining the center of mass, one tenth of the samples that are close to the center of mass was selected as a subset of the negative subset.

The above method is used to determine the initial training subset S_1_. When we trained the first weak classifier, the training dataset was classified, and the misclassified subset S_incor1_ and S_1_ were combined to construct the training subset S_2_, which was used to train the next classifier. Therefore, the training subset S_i_ is defined as:

24 $${{\rm{S}}}_{{\rm{i}}}={{\rm{S}}}_{{\rm{pos}}}\cup {{\rm{S}}}_{{\rm{neg}}1}\cup {{\rm{S}}}_{{\rm{incorr}}({\rm{i}}-1)}\cup {{\rm{S}}}_{{\rm{incorr}}({\rm{i}}-2)\ldots }\cup {{\rm{S}}}_{{\rm{incorr}}1}$$

### Classifier performance estimation

To evaluate the classification performance of the classifier, two indices are adopted, namely, the prediction accuracy and position deviation.

The prediction accuracy was defined as the percentage of the correct mature miRNA and total mature miRNA. For N pre-miRNA sequences, there are M mature miRNA candidate sequences in the ith sequence. Assuming that T (s) is the true mature miRNA and P (T) is the true mature miRNA, the prediction accuracy, Acc, can be described as:

25 $${\rm{S}}({\rm{i}})=\{\begin{array}{c}1\,{\rm{P}}({\rm{s}})={\rm{T}}({\rm{t}}),{\rm{s}}\in {\rm{M}},{\rm{t}}\in {\rm{M}}\\ 0\,{\rm{P}}({\rm{s}})\ne {\rm{T}}({\rm{t}}),{\rm{s}}\in {\rm{M}},{\rm{t}}\in {\rm{M}}\end{array}\},\,{\rm{i}}\in {\rm{N}}$$

26 $${\rm{Acc}}=\frac{{\sum }_{{\rm{i}}=1}^{{\rm{N}}}\,{\rm{s}}({\rm{i}})}{{\rm{N}}}$$

The average position deviation (APD) is the absolute value of the difference between the predicted mature miRNA position and the true mature miRNA position. It is defined as follows:

27 $${\rm{APD}}=\frac{{\sum }_{{\rm{i}}=1}^{{\rm{N}}}\,|{{\rm{t}}}_{{\rm{i}}}-{{\rm{s}}}_{{\rm{i}}}|}{{\rm{N}}}$$

where t_i_ is the nucleotide position of the ith pre-miRNA, and s_i_ is the position of the ith predicted mature miRNA.

## Results and Discussion

### Comparison of the structured-sequence-based and sequence-based methods

To investigate the classifier performance of the structured-sequence-based characters and sequence-based characters, we designed the mature miRNA identification method based on sequence-based characters. Taking P5_5 as an example, the two methods are compared in Tables 2–5:

**Table 2:** The position deviation predicted accuracy of the first candidate of sequence-based classifier.

DS	±0 nt	±1 nt	±2 nt	±3 nt	±4 nt	±5 nt	Total
Test 1	0.17	0.18	0.10	0.09	0.04	0.04	0.67
Test 2	0.64	0.09	0.13	0.04	0.04	0.01	0.95

**Table 3:** The position deviation predicted accuracy of the top five candidates of the sequence-based classifier.

DS	±0 nt	±1 nt	±2 nt	±3 nt	±4 nt	±5 nt	Total
Test 1	0.46	0.24	0.07	0.08	0.04	0.02	0.98
Test 2	0.81	0.12	0.03	0.01	0.01	0.01	0.99

**Table 4:** The position deviation predicted accuracy of the first candidate of the structured-sequence-based classifier.

DS	±0 nt	±1 nt	±2 nt	±3 nt	±4 nt	±5 nt	Total
Test 1	0.30	0.23	0.16	0.11	0.07	0.03	0.90
Test 2	0.60	0.10	0.09	0.08	0.01	0.02	0.90

**Table 5:** The position deviation predicted accuracy of the top five candidates of the structured-sequence-based classifier.

DS	±0 nt	±1 nt	±2 nt	±3 nt	±4 nt	±5 nt	Total
Test 1	0.72	0.92	0.94	0.97	1.00	1.00	1.00
Test 2	0.93	0.97	1.00	1.00	1.00	1.00	1.00

As shown in Tables 2–5, taking training set 1 as an example, we compared two classifiers that are trained based on sequence-based characters and structured-sequence-based characters: the first candidate prediction accuracy are 17% and 30%, respectively, the latter being 13% higher than the former. The position deviation predicted accuracy with the 5 nt position deviation was 67% and 90%, respectively. The latter is 33% higher than the former. The position deviation predicted accuracy of the top five candidates are 46% and 72% respectively, as the latter increased by 26%. The predicted accuracy with the 5 nt position deviation is 98% and 100%. Therefore, according to various indicators, the performance of the classifier based on the structured-sequence-based characters is greatly improved.

**Figure 5:**
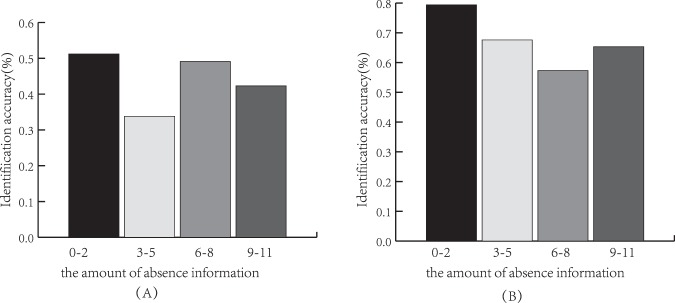
The relationship between the amount of absence information and identification accuracy. (**A**) The relationship between the amount of absence information and identification accuracy of the first candidate. (**B**) The relationship between the amount of absence information and identification accuracy of the top five candidates.

To investigate the efficiency of our tool as it varies in the presence of polynucleotide regions in the bulge of the pre-miRNAs that form the spacer in the pre-miRNA coding frame, we test it based on two test datasets. The components of bulge (absence information, “−”) were counted, and the relationship between the amount of absence information and identification accuracy are shown in Fig. 5 and Table 6.

**Table 6:** The relationship between the amount of absence information and the identification accuracy.

The amount of absence information	0–2	3–5	6–8	9–11
Accuracy based on the first candidate	51.2%	33.8%	49.1%	42.3%
Accuracy based on the top five candidates	79.4%	67.6%	57.3%	65.3%

When the amount of absence information is 0–2, the identification accuracy of the first candidate is 51.2%, and it is the highest. When the amount of absence information is 3–5, the identification accuracy of the first candidate is 33.8%, and it is the lowest. Overall, the identification accuracy decreased as the amount of absence information increased. This result can be analyzed in two ways. On the one hand, it illustrates that the identification of our tool can be affected by the amount of absence information, and with the amount of absence information increasing, the identification accuracy decreased. On the other hand, it indicates that the capture of biological characters were affected by the amount of absence information, and with the amount of absence information increasing, biological characteristics become less typical.

### Comparison of the feature selection methods

We examined three feature selection algorithms: the information gain algorithm, chi-square statistics and the relief method. First, we used all feature sets to train the classifier to obtain the accuracy of the position offset prediction of the first candidate. Then, we used these three algorithms to filter feature sets. The information gain method sorts features according to the information gain, whereas the chi-square statistic method provides a measure of the correlation between features and the categories of measurements. The relief method sorts the features according to the sample weight. According to the results of the algorithms, features that contribute a value of less than zero are deleted, and then the feature selection algorithm and the feature set are confirmed.

The position deviation predicted accuracy of the first candidate based on all features is shown in Table 7, and the position deviation predicted accuracy of the first candidate based on the chi-square and relief algorithms is shown in Tables 8 and 9. The position deviation predicted accuracy of the first candidate based on IG is shown in Table 10.

**Table 7:** The position deviation predicted accuracy of the first candidate based on all features.

DS	num	±0 nt	±1 nt	±2 nt	±3 nt	±4 nt	±5 nt	Total
Test 1	115	0.24	0.18	0.16	0.08	0.05	0.08	0.79
Test 2		0.48	0.31	0.08	0.05	0.02	0.01	0.95

**Table 8:** The position deviation predicted accuracy of the first candidate based on the chi-square algorithm.

DS	num	±0 nt	±1 nt	±2 nt	±3 nt	±4 nt	±5 nt	Total
Test 1	100	0.12	0.24	0.14	0.08	0.04	0.07	0.71
Test 2		0.36	0.18	0.09	0.06	0.05	0.03	0.77
Test 1	88	0.09	0.26	0.18	0.12	0.09	0.02	0.76
Test 2		0.28	0.21	0.16	0.05	0.06	0.02	0.78

**Table 9:** The position deviation predicted accuracy of the first candidate based on the relief algorithm.

DS	num	±0 nt	±1 nt	±2 nt	±3 nt	±4 nt	±5 nt	Total
Test 1	105	0.13	0.21	0.13	0.12	0.06	0.02	0.66
Test 2		0.32	0.21	0.07	0.08	0.03	0.01	0.72
Test 1	90	0.08	0.23	0.21	0.11	0.08	0.04	0.75
Test 2		0.35	0.29	0.09	0.06	0.03	0.02	0.84

**Table 10:** The position deviation predicted accuracy of the first candidate based on IG.

DS	num	±0 nt	±1 nt	±2 nt	±3 nt	±4 nt	±5 nt	Total
Test 1	110	0.30	0.23	0.16	0.11	0.07	0.03	0.90
Test 2		0.59	0.32	0.02	0.04	0.03	0. 00	1.00
Test 1	96	0.19	0.25	0.27	0.16	0.08	0.02	0.97
Test 2		0.44	0.35	0.07	0.04	0.04	0.02	0.96

The position deviation predicted accuracy of the first candidate of the all features, chi-square Relief and IG algorithms were 24%, 12%, 13%, and 30%, respectively. The position deviation predicted accuracy of the first candidate with 5 nt deviation was 79%, 71%, 66% and 90%, respectively. The information gain method obtained the highest prediction performance, and when the feature subset selected 110 features, we obtained the maximum prediction precision. Compared with the all features method, the position deviation predicted accuracy of the first candidate of the two test sets were 30% and 59%, and improved 6% and 11%, respectively.

### Comparison of methods before and after using the balance algorithm

To investigate the effect of balance algorithm on the classifier performance, taking Test1 as an example, we compared the methods before and after using the balance algorithm. The position deviation predicted accuracy and APD of the first candidate and the top five candidates are shown in Tables 11 and 12, respectively.

**Table 11:** The position deviation predicted accuracy and APD of the first candidate.

Classifier	±0 nt	±1 nt	±2 nt	±3 nt	±4 nt	±5 nt	sum	APD
Before using balance algorithm	0.31	0.20	0.16	0.12	0.10	0.01	0.90	2.19 nt
after using balance algorithm	0.33	0.24	0.16	0.15	0.09	0.03	1	2.05 nt

**Table 12:** The position deviation predicted accuracy and APD of the top five candidates.

classifier	±0 nt	±1 nt	±2 nt	±3 nt	±4 nt	±5 nt	Total	APD
Before using balance algorithm	0.66	0.24	0.03	0.03	0.02	0.01	0.99	1.62 nt
after using balance algorithm	0.68	0.13	0.11	0.05	0.02	0.01	1	1.24 nt

The position deviation predicted accuracy of the first candidate is 31% and 33% before and after using the balance algorithm, respectively, and the position deviation predicted accuracy with the 5 nt position deviation are 90% and 100%. The position deviation predicted accuracy of the top five candidates is 66% and 68%, and the position deviation predicted accuracy with the 5 nt position deviation is 99% and 100%. After using the balance algorithm, the APD of the top five candidates are 1.62 nt and 1.24 nt. Therefore, from the point of view of various indicators, the performance of the classifier using the balance method has been greatly improved.

### Comparison with other methods

**Figure 6:**
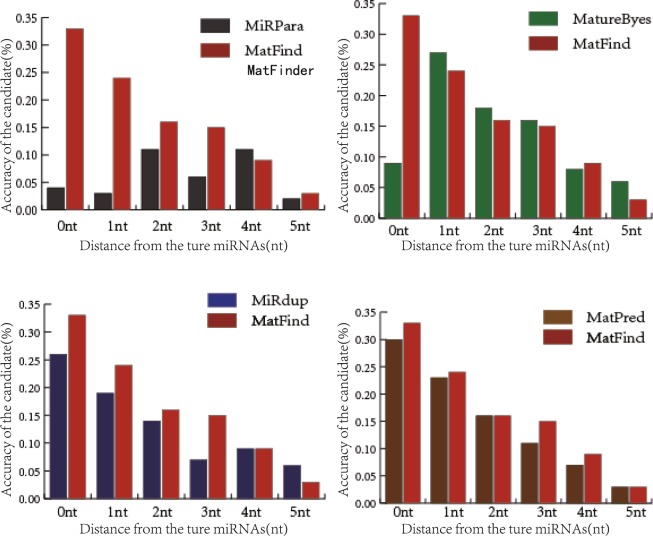
The position deviation predicted accuracy of MiRPara, MatureByes, MiRdup, MatPred and MatFinder.

Taking Test1 as an example, four methods that were developed to identify the mature miRNAs were compared with our method. The position deviation predicted accuracy of MiRPara, MatureByes, MiRdup, MatPred and MatFinder is shown in Fig. 6 and Table 13.

**Table 13:** The position deviation predicted accuracy and APD of MiRPara, MatureByes, MiRdup, MatPred and MatFinder.

classifier	±0 nt	±1 nt	±2 nt	±3 nt	±4 nt	±5 nt	Total	APD
MiRPara	0.04	0.03	0.11	0.06	0.11	0.02	0.37	5.43 nt
MatureBayes	0.09	0.27	0.18	0.16	0.08	0.06	0.84	4.65 nt
MiRdup	0.26	0.19	0.14	0.07	0.09	0.06	0.81	2.67 nt
MatPred	0.30	0.23	0.16	0.11	0.07	0.03	0.90	2.45 nt
MatFind	0.33	0.24	0.16	0.15	0.09	0.03	1	2.05 nt

The prediction accuracy of MatFinder, MiRPara, MatureByes, MiRdup and MatPred, for the first candidate was 4%, 9%, 26%, 30% and 33%, respectively. Our method, MatFinder was 29%, 24%, 7% and 3% higher than that of the other three methods. With the 5 nt position deviation, the prediction accuracy was 37%, 84%, 81%, 90% and 100%, and MatFinder was higher than the other three methods. In addition, the average position deviation is 5.43 nt, 4.65 nt, 2.67 nt, 2.45 nt and 2.05 nt, respectively. Above all, the MatFinder method is significantly superior to the other methods in the various indices.

### The performance of classifiers on all sites identification

Due to the excellent performance of MatFinder for the P5_5 site, other sites identification methods were trained based on different datasets to accomplish all sites identification. The performance of the P5_3 classifier was test based on Test1 and Test2, and the performance of the P3_3 and P3_5 classifiers were investigated using Test2 and Test3.

The position deviation predicted accuracy of the top five candidates of mature miRNA for all sites is shown in Table 14. The position deviation predicted accuracy of the P5_3, P3_5 and P3_3 classifiers was 66%, 55% and 67%, respectively. With the increase in the deviation nucleotide distance, the accuracy improved greatly. Within a 1 nt deviation, the accuracy was 92%, 89% and 84%, respectively. Within a 5 nt deviation, the recognition accuracy reached 100%, 100% and 98%, respectively.

**Table 14:** The position deviation predicted accuracy of the top five candidates of mature miRNA all sits.

Classifier	±0 nt	±1 nt	±2 nt	±3 nt	±4 nt	±5 nt	Total
P5_5	0.72	0.20	0.02	0.03	0.03	0.00	1.00
P5_3	0.66	0.23	0.08	0.01	0.00	0.00	1.00
P3_5	0.55	0.29	0.08	0.02	0.02	0.02	0.98
P3_3	0.67	0.18	0.08	0.01	0.02	0.01	0.97

## Discussion and Conclusion

MiRBase data sets consists computational predictions as well, but the computational predictions had the canonical biological characters, they will not affect our prediction results. Therefore, in our train and test dataset, we choose all the pre-miRNAs from miRBase, the experimental and computational annotations in miRBase are not separate out in our data processing.

The results show that our method, Matfinder, is superior to the other methods in identification accuracy and average position deviation. These results can be explained as follows:

The feature extraction methods based on sequence and structured-sequence were designed to investigate the biological significant of absence information. On basis of the comparison results, the structured-sequence-based method obtained the better classification results. This shows that structured-sequence-based features can represent mature miRNAs biological characteristics. It also illustrates the importance of the miRNAs structural biological characteristics. The secondary structure of miRNAs is divided into two parts, one part is the base complement of each other, the other part is isolated from the double helix region without base pairs, namely, the loop, which primarily includes: hairpin loops, the inner loops and multibranch loops. The extracted-features capture these characteristics of loops to a greater extent, so the identification accuracy is improved to a certain extent.

MatFinder is proposed based on the integration method, which is a strong classifier using AdaBoost and a weak classifier using the adjustable parameter SVM algorithm. Aiming to improve the data imbalance problem, the K-means algorithm is used to construct the center of mass of closer samples. To achieve the balanced training subsets of the data, in the process of the integrated classifier, and focus on incorrectly classified samples, the integrated results use common decision strategies of the weak classifier with different weights. This method not only solves the problem of monotone sequence diversity in mature miRNA identification but also improves the performance of the classifier.

The weak classifier is constructed using the SVM algorithm based on adjustable probability parameters. First, the SVM algorithm solves the problem of “overlearning” of the AdaBoost algorithm. Second, the SVM algorithm is based on the probability model, and the results provided five mature miRNA candidates. Importantly, in the process of integration, multiple weak classifiers based on SVM can be regulated by adjusting C and g to learn the complexity and classifier performance, which overcomes the effect of the static parameters on the performance of classifiers. The weak classifiers were trained alone with independent parameters.

In addition, we trained the P5_3, P3_5 and P3_3 classifiers based on the different biological characters. These classifiers are more accurate in identifying the first five candidates.
